# Diagnostic Efficiency of ^68^Ga-DOTATATE PET/CT as Compared to ^99m^Tc-Octreotide SPECT/CT and Conventional Morphologic Modalities in Neuroendocrine Tumors

**DOI:** 10.22038/AOJNMB.2019.39392.1263

**Published:** 2019

**Authors:** Babak Fallahi, Reyhaneh Manafi-Farid, Mohammad Eftekhari, Armaghan Fard-Esfahani, Alireza Emami-Ardekani, Parham Geramifar, Mehdi Akhlaghi, Amir Pejman Hashemi Taheri, Davood Beiki

**Affiliations:** 1 Research Center for Nuclear Medicine, Shariati Hospital, Tehran University of Medical Sciences, Tehran, Iran; 2 Department of Radiology, Shariati Hospital, Tehran University of Medical Sciences, Tehran, Iran; † These authors shared first authorship

**Keywords:** Conventional imaging, Neuroendocrine tumor, ^ 68^Ga-DOTATATE PET/CT, ^ 99m^Tc-Octreotide SPECT/CT

## Abstract

**Objective(s):**

In view of somatostatin receptor (SSR) expression on cell membranes of the majority of neuroendocrine tumors (NETs), functional imaging exploiting analogs of SSR alongside the anatomical imaging is the mainstay of this diagnostic modality. In this prospective study, we assessed and directly compared the diagnostic parameters of ^68^Ga-DOTATATE PET/CT and ^99m^Tc-Octreotide SPECT/CT, as well as CT/MRI.

**Methods:**

Twenty-five NET patients, either histologically proven or highly suspicious for NET, who were referred for Octreotide Scan were enrolled in this prospective study. They all underwent ^99m^Tc-Octreotide SPECT/CT and then ^68^Ga-DOTATATE PET/CT. A blind interpretation was conducted for each imaging as well as for the previously obtained conventional imaging (CT or MRI). The patient-based and lesion-based analysis were conducted and the results of the three modalities were compared. The histopathologic confirmation or follow-up data were considered as the gold standard. Also, the impact of ^68^Ga-DOTATATE PET/CT on the patient’s management was assessed.

**Results:**

Overall, 77 lesions in 14 patients, 135 in 19 and 86 in 16 were detected on ^99m^Tc-Octreotide SPECT/CT, ^68^Ga-DOTATATE PET/CT and CT/MRI, respectively. On patient-based analysis, the sensitivity was 65%, 90% and 71% for ^99m^Tc-Octreotide SPECT/CT, ^68^Ga-DOTATATE PET/CT and CT/MRI, respectively. Also, the specificity was 80%, 80% and 75% for ^99m^Tc-Octreotide SPECT/CT, ^68^Ga-DOTATATE PET/CT and CT/MRI, respectively. The correlation between ^68^Ga-DOTATATE PET/CT and ^99m^Tc-Octreotide SPECT/CT results was significant (=0.02; kappa value=0.57), no correlation, however, was depicted with CI (=0.07; kappa value=0.35). On lesion-based analysis, ^68^Ga-DOTATATE PET/CT found more organs (=0.02) and lesions (=0.001) in comparison with ^99m^Tc-Octreotide SPECT/CT and also more lesions in comparison with CT/MRI (=0.003). In addition, comparing with ^99m^Tc-Octreotide SPECT/CT and CT/MRI, ^68^Ga-DOTATATE PET/CT revealed more data in 44% and 36% of the patients, resulting in management modification in 24% and 20%, respectively.

**Conclusion:**

Comparing with ^99m^Tc-Octreotide SPECT/CT and CT/MRI, ^68^Ga-DOTATATE PET/CT provided more sensitivity and specificity in patients with NETs showing more involved organs as well as tumoral lesions. Also, ^68^Ga-DOTATATE PET/CT led to change of management in up to one-fourth of the patients, especially in a sub-group re-evaluated for recurrence.

## Introduction

Neuroendocrine tumors (NETs) are rare slow-growing malignancies (1, 2) originating from the hormone-producing neuroendocrine system (3). The incidence has remarkably increased, recently (4, 5). Signs and symptoms of excessive hormone secretion are not clearly present in all patients, leading to delayed diagnosis (2). Hence, NETs are commonly detected in advanced, inoperable, or metastatic status (4, 6). Although NETs are generally non-aggressive, the metastasis is a frequent finding (2). Liver, lymph nodes and bone are the main metastatic target organs (7).

Multitudes of morphological and functional imaging modalities, such as sonography, CT, MRI, and scintigraphy have been employed for staging, re-staging and evaluation of response to therapy (1, 4, 6). The morphological imaging such as multi-detector CT with contrast medium (CM) provides rapid imaging and dynamic evaluation of CM which improves image resolution and leads in the detection of small NET lesions (8). Also, whole-body diffusion-weighted magnetic resonance Imaging (DW-MRI) without CM administration has been utilized for the evaluation of metastasis, despite the fact that the procedure is time-consuming and expensive (9). 

Somatostatin receptors (SSRs) are found on cell membranes in the majority of the NETs (2). Five subtypes of SSRs have been discovered in various body cells as well as tumoral lesions (10, 11). However, SSR2 and SSR5 are expressed in 70-90% of NETs (12). Initial functional imaging with SSR-analogues was performed exploiting ^111^In-DTPA Pentetreotide, which remained the method of choice for more than two decades (2). However, low-quality images, higher cumulative dose, long imaging protocol, etc. were always problematic (9, 10, 13). 

Superior sensitivity and better resolution provided by PET and high avidity of ^68^Ga-DOTA-conjugated-peptides to SSRs revolutionized NET imaging (11). By virtue of ^68^Ga-DOTA-conjugated-peptides PET, not only image quality is enhanced but also the duration of the imaging procedure and effective dose are reduced (10, 11). Of note, a wide array of tumoral lesions express these receptors and can be visualized by ^68^Ga-DOTA-conjugated-peptides PET scan (10). 

Safety and efficiency of ^68^Ga-DOTA-conjugated-peptides PET scan has also been demonstrated (14). In a recent meta-analysis, the estimated sensitivity of 90.9% and specificity of 90.6% have been reported for ^68^Ga-DOTATATE PET/CT (1). In addition, its impact on management alteration has been documented in a substantial number of patients (2, 15, 16). Ultimately, FDA approved NETSPOT® as the first kit for the preparation of ^68^Ga-DOTATATE for NET imaging in both adults and children, in June 2016 (14). 

Although different procedures have been employed for the treatment of NET, the surgical removal of the tumor can be curative in these patients (17). It is clear that an accurate pre-therapy delineation of NET is imperative to successfully treat or control the disease. It seems that ^68^Ga-DOTATATE PET/CT is replacing the conventional scintigraphic method in the majority of centers. In this prospective study, we evaluated the diagnostic parameters of ^68^Ga-DOTATATE PET/CT in comparison with ^99m^Tc-Octreotide SPECT/CT as well as conventional anatomic methods. Cost difference, however, still remains of concern in many centers throughout the world. 

## Methods

***Study population***

The study was approved by the ethics committee of Tehran University of Medical Sciences (IR.TUMS.MEDICINE.REC.1395.858) and written informed consent was obtained from all patients following a detailed oral and written explanation. 

Twenty-five patients, either histologically proven or highly suspicious cases with future histological confirmation, were enrolled in this prospective study, from March to December 2017. The mean age was 52.0±14.4 y (range: 31-76 y) consisting of 36% (9/25) females and 64% (16/25) males. All patients were referred to our department for ^99m^Tc-Octreotide SPECT/CT imaging. Patients were not enrolled in this study in case of pregnancy, breastfeeding, or second malignancy. Moreover, the study was postponed more than 4 weeks following cessation of long-acting Octreotide, five weeks after the conclusion of chemotherapy, and six weeks from any surgical treatment. Also, patients were excluded if the time interval between the imaging modalities exceeded 30 days or there was any other therapeutic intervention between procedures.

Twenty-four percent (6/25) were referred for initial staging, 48% (12/25) for re-staging, 24% (6/25) for response to therapy, and 4% (1/25) for localizing the primary tumor. The pathology confirmed carcinoid tumor in 32% (8/25), medullary thyroid cancer in 12% (8/25), gastrinoma in 8% (2/25), and non-specific NET in 44% (11/25). One of the patients (4%) was referred with extra-cranial ACTH syndrome in whom all modalities failed to localize the tumor and pathological confirmation was not possible. Ki-67 was available in only 15 patients with mean 6.8% (median=5%). Ki-67 was less than 40% in all cases. However, in 9 patients with no available Ki-67, ^68^Ga-DOTATATE PET/CT depicted at least one SSR-avid lesion. Details regarding age, NET type, Ki-67, and indication of referral are illustrated in Table 1.

***Radiopharmaceutical preparation***

Tc-99m and Ga-68 were respectively obtained from commercially ^99^Mo/^99m^Tc (PARSTEC II) and ^68^Ge/^68^Ga (PARS-GalluGEN) generators, which are locally available in the country form Pars Isotope Company, Tehran, Iran. The freeze-dried kits of HYNIC-Tyr^3^-Octreotide (HYNIC-TOC) and DOTA-Tyr^3^-Octreotate (DOTATATE) were also provided from above-mentioned company. The radiopharmaceutical preparation (^99m^Tc-Octreotide/^68^Ga-DOTATATE) and quality controls were carried out according to the standard protocols provided by the kit manufacturer. 

***Patient preparation, image acquisition and reconstruction***

^99m^ ***Tc-Octreotide SPECT/CT***

^99m^Tc-Octreotide SPECT/CT was performed following intravenous administration of approximately 925 MBq (25 mCi) ^99m^Tc-Octreotide, considering maximum 10% error in administered activity. Planar acquisition started 3 hours after injection. Subsequently, after voiding, SPECT/CT was performed, consisting of at least two acquisition beds from the skull-base to the upper-thigh region, encompassing the whole pelvic cavity. Planar whole-body and SPECT/CT studies were obtained using a dual-head gamma camera (Siemens-Symbia T1), equipped with a low-energy high resolution (LEHR) parallel-hole collimator. Whole body acquisition parameters were 15cm/min table speed, matrix size of 256×1024 and 140 keV energy window with 10% width. Tomographic acquisition was performed in a non-circular orbit using 7 sec/frame for ninety frames and 128×128 matrix size with zoom of 1. CT scan was obtained for anatomical correlation and attenuation correction without oral or intravenous contrast medium injection (spatial resolution 3mm, 110 kV, 60-80 mAs, the average scanning time of 12 seconds).

^99m^Tc-Octreotide SPECT/CT images were reviewed by two experienced nuclear medicine specialists who were aware of the clinical information, but not the result of newly reported CT or MRI. In case of discordant reports, a third nuclear medicine specialist was consulted and the final interpretation was based on consensus. We used Krenning scoring system for interpreting ^99m^Tc-Octreotide SPECT/CT. This system attributes score 0 for no abnormality, 1 for faint uptake in the tumor, 2 for clear uptake in the tumor but less than in the liver, 3 for uptake greater in the tumor than in the liver, and 4 for uptake much greater in the tumor than in the liver. A score ≥2 was labeled positive. We also considered the corresponding morphological finding on concomitant CT scan for the final diagnosis (18). The data were recorded according to the number of involved organs and the number of lesions detected per organ. In case of conglomerated lymph nodes, when determination of involved lymph nodes was individually impossible, they were considered as one mass lesion in the final analysis. SPECT, CT, and fusion SPECT/CT data were viewed and analyzed using Syngo software (Siemens Medical Systems).

^68^ ***Ga-DOTATATE PET/CT***

Whole-body scan, from skull-base to mid-thigh, was performed 60 minutes after approximately 185 MBq (5 mCi) ^68^Ga-DOTATATE bolus IV injection using a Siemens Biograph 6 TruePoint PET/CT scanner. Again, less than 10% error for administered activity was considered acceptable. In case of equivocal or unclear findings, extra bed-positions were obtained accordingly. All images were reconstructed identically using the ordered-subsets expectation maximization (OSEM) algorithm with two iterations and 21 subsets followed by a post-reconstruction smoothing with a Gaussian filter (4.0 mm FWHM). Native CT scan was performed applying following parameters: 120–130 mA, 0.6 s per rotation, 5.0 mm reconstructed section thickness, 0.5 mm overlap, 512×512 matrix, and pitch index 1.3. 

PET/CT images were interpreted using the same protocol as ^99m^Tc-Octreotide SPECT/CT studies. Moreover, morphologic findings seen on corresponding non-diagnostic CT images were utilized to categorize the lesions, especially lymph nodes, with borderline activity as malignant or begin. In addition, maximum standardized uptake value (SUV_max_) was measured by drawing region of interest (ROI) over corresponding areas. The analysis was performed using Syngo software-TrueD (Siemens Medical Systems).

***Conventional imaging***

All the participating patients had already undergone a CT, MRI or both of these diagnostic procedures during the course of their disease before inclusion in this study. It was decided not to subject the patients to extra radiation and/or contrast medium administration. Nineteen (76%) patients had CT imaging (56% with and 20% without CM), 1 (4%) had MRI with CM, 2 (8%) had MRI and contrast-enhanced CT, and 3 (12%) had both CT and MRI with CM. All images were collected and reviewed by an expert radiologist who was aware of the clinical information as well as previous radiology reports, but blind to ^99m^Tc-Octreotide SPECT/CT or ^68^Ga-DOTATATE PET/CT imaging results. 

***Standard of the truth***

**Figure 1:**
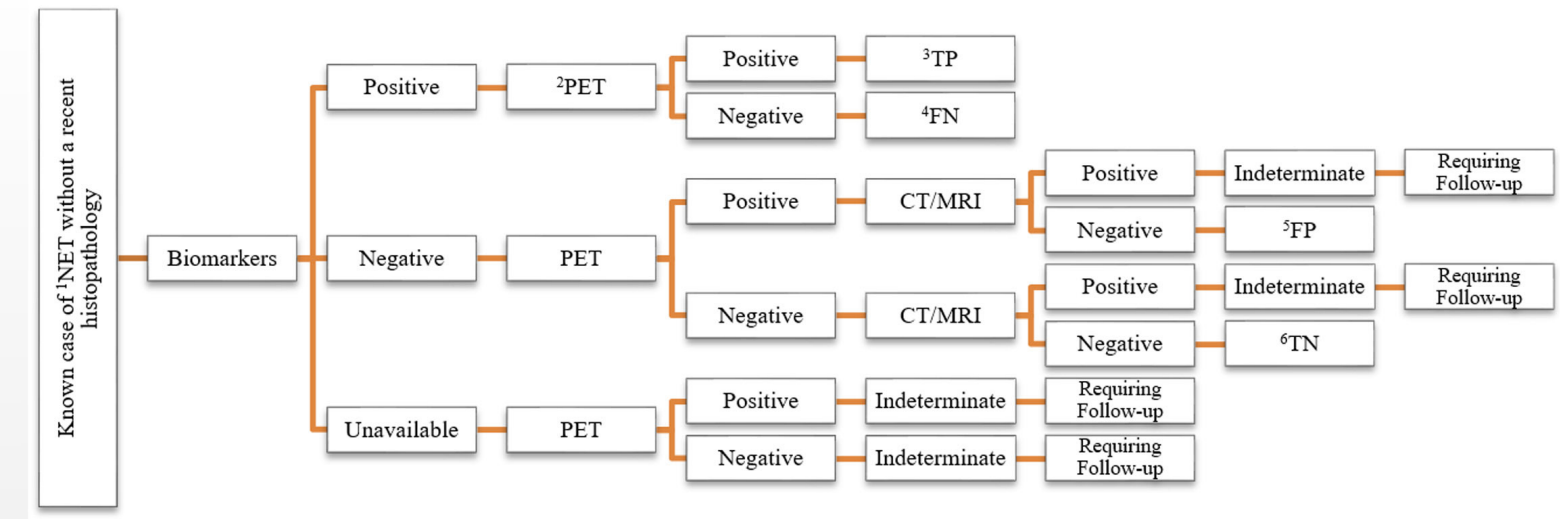
The algorithm employed in defining true and false findings. In case of an indeterminate finding the result was correlated with histopathology. ^1^Neuroendocrine Tumor; ^2^PET/CT with ^68^Ga-DOTATATE; ^3^True Positive; ^4^False Negative; ^5^False Positive; ^6^True Negative

In order to define true and false findings, detected lesions were labeled as malignant or benign. For equivocal findings the categorization was based on consensus. Thereafter, patients were followed up to 10 months to determine the false and true findings. If there was a histopathology of the detected lesions, they conveniently were defined true positive (TP) or true negative (TN). When a histopathology of only one detected lesion was available, in case of radio-tracer uptake similarity, they were also considered malignant and TP. The challenge, however, was attributed to metastatic lesions where the primary tumor was previously excised and no reference uptake was available for comparison. We designed an approach to precisely categorize the lesions using other clinical and follow-up data. The algorithm is illustrated in Figure 1. In summary, in case of an elevated biomarker, lesions were considered TP or false negative (FN) based on positive or negative ^68^Ga-DOTATATE PET/CT result, respectively. If the pertinent biomarker was not available, the lesions were considered indeterminate. If both biomarker and CT/MRI result were negative, lesions were considered false positive (FP) or TN, again, based on positive or negative ^68^Ga-DOTATATE PET/CT result, respectively. Finally, if the biomarker was negative and a definite lesion or an equivocal one was detected on CT/MRI, the lesion was classified as indeterminate. Indeterminate lesions were further followed for histologic confirmation. When the lesions remained indeterminate, the patients were excluded from the analysis. Table 2 provides corresponding biomarkers regarding the attributed NET evaluated in our patients. 

***Statistical analysis***

Numerical data were presented as medians and means±SD. Data which did not show a normal distribution on Kolmogorov–Smirnov test were compared using the nonparametric tests. A p-value lower than 0.05 was considered significant. Statistical analysis was conducted with dedicated software (SPSS 24.0; IBM Corp., Armonk, NY).

In the patient-based analysis, the final result (i.e. positive vs. negative study for the presence of any tumoral lesion) was considered for the comparison between the procedures regardless of the number of detected lesions or involved organs. While in organ-based and lesion-based analyses, the number of detected lesions and involved organs were compared, respectively. Based on the binary final results (positive/negative), we analyzed the agreement between two different modalities using kappa agreement analysis. We employed a Chi-square test to compare the potential of each modality in the detection of abnormal results. Wilcoxon test was utilized to depict any superiority of ^68^Ga-DOTATATE PET/CT in the localization of higher numbers of involved organs or abnormal lesions. 

## Results

^99m^Tc-Octreotide SPECT/CT was positive in 56% (14/25) of the patients showing a total number of 77 lesions detected by this method. Diagnostic parameters, false and true findings are demonstrated in Table 3. It is of note that ^99m^Tc-Octreotide SPECT/CT and ^68^Ga-DOTATATE PET/CT localized a suspicious lesion, precaval region, posterior to the uncinate process and pancreatic head, in one patient with elevated ACTH level, which was subsequently negative by tissue examination; while no other lesion was detected by multi-modality imaging despite the presence of highly elevated and rising values of ACTH. Although incisional sampling error may be a possible explanation for this discrepancy, this patient was classified in FP group.

Moreover, detected lesions were also analyzed according to the location and involved organs as follows: 1) neck, lung, and mediastinum, 2) pancreas, 3) liver, 4) abdominal lymph nodes, 5) gastrointestinal loop and 6) miscellaneous parts of the body. The true or false findings, as well as diagnostic parameters, were evaluated regarding each category. The sensitivity of ^99m^Tc-Octreotide SPECT/CT was highest for pancreatic lesions (83.3%), while the test was positive in only half of the involved abdominal lymph nodes (50%) and one-fourth of the neck and thoracic lesions (25%). 

When diagnostic parameters for the detection of the lesions were evaluated based on the location of the lesions, ^68^Ga-DOTATATE PET/CT detected NET in 76% (19/25) of the patients representing a total number of 135 lesions while ^68^Ga-DOTATATE PET/CT detected all lesions in the neck and thoracic regions (sensitivity=100%). The lowest sensitivity was attributed to liver and pancreas (83.3%). 

Finally, CT/MRI detected a total number of 86 neuroendocrine tumoral lesions in 66% (16/25) of the patients. The sensitivity for the detection of pancreatic lesions was only 33.3%; while, it was 83.3% for gastrointestinal tract lesions.

***Comparative data***

Regarding patient-based analysis, ^68^Ga-DOTATATE PET/CT showed a significant agreement with ^99m^Tc-Octreotide SPECT/CT results (=0.02; kappa value=0.57), but not with CT/MRI (=0.07; kappa value=0.35).

**Figure 2:**
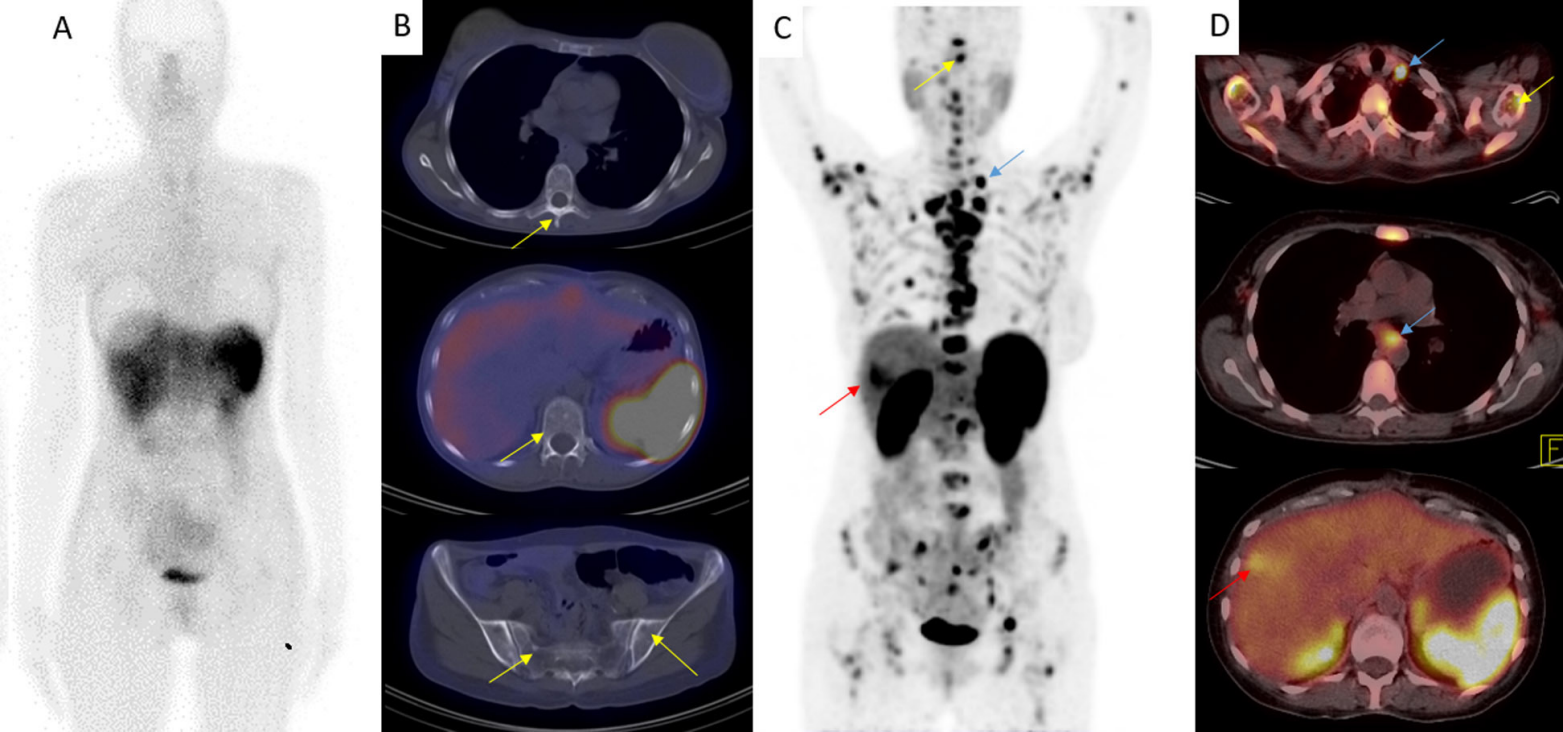
A 40-year-old female with history of metastatic carcinoid tumor, undergone partial pancreatectomy and liver transplantation two years ago. She was evaluating for increasing levels of chromogranin A. (A) Planar and (B) transaxial ^99m^Tc-Octreotide SPECT/CT showed physiologic radiotracer distribution without any abnormal focus of uptake. Lytic/sclerotic lesions were apparent on low-dose CT (yellow arrows). (C) Whole body and (D) transaxial ^68^Ga-DOTATATE PET/CT revealed widespread bone metastases (yellow arrows), as well as metastatic lymph nodes (blue arrows) in the left supraclavicular and mediastinal regions. Also, evidence of fatty liver with a focal spared parenchyma (red arrow) is visualized

^68^Ga-DOTATATE PET/CT detected more involved organs (=0.02) and also more lesions (=0.001) in comparison with ^99m^Tc-Octreotide (Figure 2). ^68^Ga-DOTATATE PET/CT detected more involved organs and lesions in 8 and 15 patients, respectively. However, all tumoral lesions depicted on ^99m^Tc-Octreotide SPECT/CT were also positive on ^68^Ga-DOTATATE PET/CT. In addition, regarding the number of organs, the difference between ^68^Ga-DOTATATE PET/CT and CT/MRI was not statistically significant (=0.15). There were more involved organs on ^68^Ga-DOTATATE PET/CT in 8 patients. While CT/MRI localized more involved organs in 3 and there was the same number of involved organs in 14 patients-including normal studies. On the other hand, ^68^Ga-DOTATATE PET/CT found significantly more lesions than CT/MRI did (=0.003). There were more lesions identified in 18 patients on ^68^Ga-DOTATATE PET/CT, while CT/MRI found more lesions in only 2 patients. 

**Figure 3:**
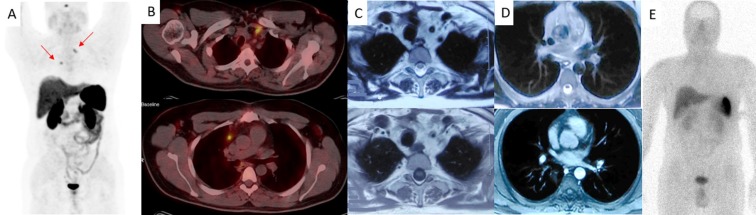
A 45-year-old male with medullary thyroid carcinoma, undergone total thyroidectomy and bilateral cervical and mediastinal lymph nodes dissection. He was re-evaluating for the increasing calcitonin levels. (A) Maximum intensity projection (MIP) and (B) transaxial ^68^Ga-DOTATATE PET/CT revealed two SSR-positive lymph nodes in the left supraclavicular and right prevascular regions (red arrows). (C) Transaxial (T1/T2) and (D) (T1/contrast enhanced) MRI revealed only non-specific post-surgical changes. (E) Planar ^99m^Tc-Octreotide SPECT/CT showed the physiological distribution of the radiotracer. Subsequent histological findings confirmed tumoral involvement of the detected lymph nodes

Furthermore, the impact of additional data provided by ^68^Ga-DOTATATE PET/CT influencing patient management was assessed namely modification from surgery to systemic therapy (long-acting Octreotide or chemotherapy) and vice versa or change in surgical approach. Comparing ^68^Ga-DOTATATE PET/CT and ^99m^Tc-Octreotide SPECT/CT, the former provided more information in 44% (11/25) of the patients regarding the number of organs or lesions, leading to management alteration in only 24% (6/25), one out of six (16.7%) in the staging and five out of twelve (41.7%) in the re-staging group. Likewise, ^68^Ga-DOTATATE PET/CT demonstrated more involved organs or lesions in 36% (9/25) of patients in comparison with CT/MRI, resulting in management alteration in only 20% (5/25), one out of six (16.7%) in the staging and four out of twelve (33.3%) in the re-staging group. It worth noting that ^68^Ga-DOTATATE PET/CT correctly detected at least one tumoral lesion in 5 out of 7 false negative ^99m^Tc-Octreotide SPECT/CT and in 4 out of 6 false negative CT/MRI studies (Figure 3).

**Table 1:** Details regarding age, NET type and indication for referral

**Neuroendocrine tumor type**	**Gender**	**Age**	**Ki-67**	**Indication**
Gastroentheropancreatic	Male: 12 (75%)	54.9±14.4	Available in 11 Mean: 4.7% Median: 4.5%	Staging: 6
				Restaging: 7
	Female: 4 (25%)			DPS: -
				FUE: 3
Bronchial Carcinoid	Male: 2 (50%)	35.2±6.5	Available in 3 Mean: 3.6% Median: 3.0%	Staging: -
				Restaging: 2
	Female: 2 (50%)			DPS: -
				FUE: 2
Medullary Thyroid Carcinoma	Male: 1 (33%)	51.0±5.2	Available in 0	Staging: -
				Restaging: 3
	Female: 2 (67%)			DPS: -
				FUE: -
Other	Male: 1 (50%)	64.5±12.0	Available in 1 40%	Staging: -
				Restaging: -
	Female: 1 (50%)			DPS: 1
				FUE: 1
Total	Male: 16 (64%)	52.0±14.4	Available in 15 Mean: 6.8% Median: 5.0%	Staging: 6 (24%)
				Restaging: 12 (48%)
	Female: 9 (36%)			DPS: 1 (4%)
				FUE: 6 (24%)

DPS: Detection of the primary site; FUE: Follow-up evaluation

**Table 2:** Certain biomarkers regarding the attributed neuroendocrine tumor (NET)

**Neuroendocrine tumor**	**Chromogranin A**
Carcinoid tumor	5-hydroxyindoleacetic acid
Gastrinoma	Gastrin
Medullary thyroid cancer	Calcitonin
Insulinoma	Insulin and C-peptide

**Table 3:** Diagnostic parameters, false and true findings of different imaging modalities according to patient-based analysis

**Type of the Procedure**	**Results**				**Sensitivity**	**Specificity**	**PPV**	**NPV**	**Accuracy**
	**TP**	**TN**	**FP**	**FN**					
^99m^Tc-Octreotide SPECT/CT	13 (52%)	4 (16%)	1 (4%)	7 (28%)	65.0%	80.0%	92.8%	36.4%	68.0%
^68^Ga-DOTATATE PET/CT	18 (72%)	4 (16%)	1 (4%)	2 (8%)	90.0%	80.0%	94.7%	66.7%	88.0%
CT/MRI	15 (60%)	3 (12%)	1 (4%)	6 (24%)	71.4%	75.0%	93.7%	33.3%	72.0%

TP: True Positive; TN: True Negative; FP: False Positive; FN: False Negative; PPV: Positive Predictive Value; NPV: Negative Predictive Value

**Figure 4:**
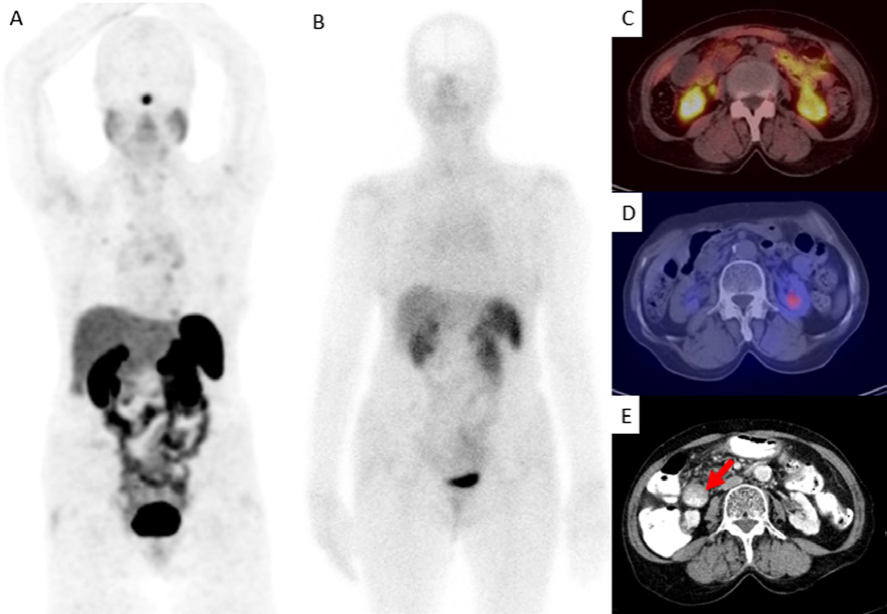
A 73-year-old woman with NET of duodenum. (A) Maximum intensity projection (MIP) and (C) transaxial ^68^Ga-DOTATATE PET/CT shows physiologic activity in the duodenum without any focal abnormality. She was referred for initial staging. (B) Planar and (D) transaxial ^99m^Tc-Octreotide SPECT/CT illustrates physiological distribution of the radiotracer. (E) Transaxial CT with intravenous and oral contrast medium shows the evidence of mass-like lesion in second part of the duodenum with mucosal thickening (red arrow)

## Discussion

NETs originate from the hormone-producing neuroendocrine system of mainly gastrointestinal (72%) and broncho-pulmonary (25%) systems and less than 5% from other parts of the body (3, 4). The incidence of NETs is augmenting in recent decades (4, 5). Due to heterogeneous signs and symptoms, as well as the different origin of NETs, the diagnosis of these tumors is still challenging (2). Significant disease-related mortality and morbidity (2) indicate a need for accurate diagnostic modalities in decision making and choosing an optimum treatment plan. SSRs functional imaging has proved to be of indisputable value in NET evaluation. Recently approved modality, ^68^Ga-DOTA-conjugated peptides PET/CT has revealed a multitude of advantages (11, 14) and it is gradually substituting the previous standard ^111^In-Pentetreotide scintigraphy. This modality is not widely available in all nuclear medicine centers. In this study, we directly compared the results of ^68^Ga-DOTATATE PET/CT with routinely performed conventional ^99m^Tc-Octreotide scan along with anatomical imaging (CT and/or MRI) to clarify if it provides diagnostic superiority or demonstrates additional clinical impact, especially in comparison to sophisticated SPECT/CT hardware and softwares currently introduced to conventional nuclear medicine. 

Different SSR-analogues show subtle, nevertheless, a different pattern of avidity to somatostatin receptors 2-5 leading to inconspicuous difference in biodistribution (4, 19). It has been documented that SUV_max_ of tumoral lesions in different organs reveals similar uptake intensity using ^68^Ga-DOTATOC and ^68^Ga-DOTATATE (20), with ^68^Ga-DOTATATE showing a considerably high affinity for SSR2 (9, 20). Furthermore, in earlier studies, comparable tracer uptake has been reported using both ^99m^Tc- and ^111^In-labeled-SSR analogues (21). Thus, we compared the results of our investigation with those of other investigations, which have been used different tracers. 

^68^ ***Ga-DOTATATE PET/CT***

The sensitivity of ^68^Ga-DOTATATE PET/CT was 90.0% in the current study, which is in accordance with other investigations. In a large retrospective study and another meta-analysis, the overall sensitivity has been reported as 87.1% and 90.9% (81.4%–96.4%, with 95% confidence interval), respectively (1, 22). The small tumor size, low SSR expression, de- or undifferentiated NETs, and presence of high physiologic uptake was reported as reasons for the false negative result (11). 

Significant physiological tracer uptake is a common finding in the uncinate process of the pancreas, related to the particular population of cells expressing SSRs (23). The mean SUV_max_ of 10.5 (range: 2.9–28.7) has been reported for this region (24). It has been suggested that every uptake in this area should be considered physiologic in the absence of any morphological change (23, 24). However, it is still regarded as a challenging issue. We also encountered a patient with focal non-homogeneous tracer uptake in the uncinate process of the pancreas, needle biopsy, however, did not reveal any evidence of malignancy.

The specificity of ^68^Ga-DOTATATE PET/CT has been reported equal to 90.6%, with 95% confidence interval of 77.8 to 96.1% (1) and even higher approaching to 100% reported in some investigations (18, 22). In our study, the specificity was 80% which was slightly lower, although within the reported range which is most likely attributed to the characteristics of the population enrolled in our study. Most of the patients referred to our department were clinically complicated with controversial pathology. The majority were not disease-free. Thus, the number of disease-free patients was small, reflected as a potential bias up on our study. Therefore, Negative Predictive Value (NPV) was significantly lower in comparison with prior investigations (57.1% vs. 61.1%-96.2%) (22). 

Although the uptake of ^68^Ga-DOTA-conjugated peptides is not limited to NETs, the Positive Predictive Value (PPV) was 94.7%, which was similar to the previously reported range for primary as well as metastatic lesions (90%-100%) (9, 22, 25). The superiority of PET/CT imaging along with the quantification option could be the explanation. It has been shown that SUV_max_ of the malignant tumors is remarkably higher than benign lesions (20). This emphasizes the advantage of ^68^Ga-DOTATATE PET/CT in more accurately marking the detected lesions as tumors.

^68^ ***Ga-DOTATATE PET/CT vs.*** ^99m^ ***Tc-Octreotide SPECT/CT***

The overall study results showed an agreement between ^68^Ga-DOTATATE PET/CT and ^99m^Tc-Octreotide SPECT/CT (p=0.02; kappa value=0.57). This was not beyond expectations since both modalities employ SSRs imaging. However, as it has been demonstrated (1), ^68^Ga-DOTATATE PET/CT showed significantly more lesions as well as involved organs. 

The sensitivity of the ^99m^Tc-Octreotide SPECT/CT in our study was 65.0% which is slightly lower compared with other reports (67%-100%) (26), which was also through concerning specificity (80% vs 92%), PPV (92.8% vs. 98%), and NPV (36.4% vs. 47%), as compared to other investigations (27). The complicated nature of the patient population could be the explanation for the lower s detected in our survey. 

The sensitivity of the ^99m^Tc-Octreotide SPECT/CT was dramatically low for tumors of the neck and thoracic regions (25%). We assume that this finding is most likely due to low detectability of ^99m^Tc-Octreotide scan in cases of MTC. Likewise, it has been shown that the sensitivity of ^99m^Tc-Octreotide scan is compromised in these patients (28). It has been shown that the sensitivity of the ^111^In-DTPA-Octreotide scan as well as ^68^Ga-DOTA-conjugated peptide PET/CT differs for different cases of NETs, mostly depending on the type and the size of the tumor, as well as the level of SSRs expression (3, 10). 

The sensitivity for detection of Insulinoma, for example, has been reported 50-70% due to the nature of smaller size and low expression of SSR. While, the sensitivity is over 80% for gastroenteropancreatic NETs (11). Also, the level of Ki-67 index affects the sensitivity. The poor differentiation of NET can cause false negative results on ^68^Ga-DOTA-conjugated peptides PET/CT (29) and tumors with higher Ki-67 index (specially>55%) are better visualized on ^18^F-FDG PET/CT rather than with SSR-analogues (13). 

It has been shown that ^68^Ga-DOTATATE PET can detect tumoral lesions in some patients with negative or equivocal ^111^In-DTPA-Octreotide scan (1), indicating the higher efficacy of ^68^Ga-DOTATATE PET/CT in the detection of involved organs or number of lesions. Likewise, in our study, ^68^Ga-DOTATATE PET/CT not only localized more lesions, nearly detected all lesions seen on ^99m^Tc-Octreotide SPECT/CT, except for one adrenal adenoma, which was proved to be benign in the follow-up evaluation. These findings could be explained by the better spatial resolution of PET allowing detection of smaller lesions, and also substantially higher affinity of TATE to SSR2 receptors (9, 20). On the contrary, Krausz et al. have reported a case with a positive liver lesion on ^111^In-DTPA-Octreotide (SPECT or SPECT/CT) and negative on ^68^Ga-DOTANOC PET/CT suggesting the difference in kinetics of the tracers as the explanation (26). False positive results which can be seen in SPECT only Octreotide conventional scintigraphy, might also be a potential source of misinterpretation.

Moreover, the impact of ^68^Ga-DOTA-conjugated peptides on the management of NET patients has been emphasized in several studies (2, 16, 30, 31).

In a study on 59 patients comparing In-111 octreotide and ^68^Ga-DOTATATE PET/CT, Hofman et al. have demonstrated that the latter had high management impact in 47% and moderate impact in 10% of the patients (31). Likewise, in comparison with ^111^In-pentetreotide imaging, Deppen et al. have reported that ^68^Ga-DOTATATE PET/CT showed higher sensitivity for the detection of tumoral foci and resulted in a change in clinical management of 36% of the patients. Additionally, by the exclusion of ^111^In-pentetreotide study, no remarkable negative consequences were observed (2). Moreover, the change in the clinical decision has been reported in 32.8% of 131 patients by Sadowski et al (15). In our study, by comparing with conventional ^99m^Tc-Octreotide SPECT/CT, ^68^Ga-DOTATATE PET/CT provided additional information in 44% and change of treatment in 24% of the participants. The impact on management was remarkably higher when applied for re-staging as opposed to staging sub-group (5/12 vs.1/6).

^68^ ***Ga-DOTATATE PET/CT vs. CT/MRI***

The current study failed to demonstrate an agreement between ^68^Ga-DOTATATE PET/CT and CT/MRI results (p=0.07; kappa value=0.35). This disagreement, in turn, could be due to the potential inherent difference between these two imaging modalities (functional vs. morphological), as very small primary lesions or micro-metastases undetectable on CT/MRI might express SSRs, adequate enough to be captured by PET scanner. In addition, prior surgical manipulation and distorted anatomy could be a source of false interpretation by CT/MRI (26). 

The sensitivity, specificity, NPV, PPV, and accuracy for CT/MRI were 71.4%, 75.0%, 33.3%, 93.7%, and 72.0% in our study. The sensitivity of CT and MRI for pancreatic NETs has been reported as 82% and 79%, respectively (32). Also, the overall sensitivity for whole-body DW-MRI has been reported as 72% in a prospective study (9). However, in our study, the sensitivity of CT/MRI was the lowest in the pancreas (33.3%). This, in part, is due to the small size of the lesions in this organ. 

The overall sensitivity for CT/MRI was lower in our study, likely due to the complicated nature of the patients, mostly with history of previous surgical interventions. Another factor, although to a lesser extent, might be the utilization of the patient’s prior CT and MRI studies performed differently in other centers for comparison.

It has been shown that the detection rate of lesions located in the gastrointestinal tract ‘hollow organs’ is not satisfactory with CT/MRI, reportedly 22-45% for mid-gut carcinoid and gastrinomas (26). In addition, in case of primary tumors of the small intestine, the patient-based sensitivity of 25% for contrast-enhanced CT and 89% for ^68^Ga-DOTA-conjugated peptides PET/CT has been documented (17). Also, additive value of ^68^Ga-DOTATATE PET/CT has been shown by enhancing the sensitivity (94% vs. 63%) and accuracy (87 % vs. 68 %) of primary NETs detection compared with contrast-enhanced CT alone (33). However, in the current study, the sensitivity and specificity of CT/MRI was 83.3% and 100%, respectively, for GI tract tumors, similar to ^68^Ga-DOTATATE PET/CT. The different range of sensitivity for CT/MRI could be due to the diverse nature of NETs namely tissue of origin and, more importantly, the size of the tumor. 

Positive results for malignancy had been reported in two patients on CT scan and one patient on liver-MRI, which were overlooked by ^68^Ga-DOTATATE PET/CT. In follow-up evaluation, a tumor was diagnosed in one patient in the duodenum (Figure 4). However, the other two lesions were proved to be false positive findings-one in the adrenal gland (on CT) and the other in the liver (on MRI). This reflects the higher specificity of ^68^Ga-DOTATATE PET/CT in comparison with anatomical imaging. The reason for the false negative finding on ^68^Ga-DOTATATE PET/CT could be explained by the special site of the tumor (duodenum), it’s small size, and high regional physiologic uptake (duodenal wall and pancreatic head). 

Although statistically insignificant, ^68^Ga-DOTATATE PET/CT found more involved organs. In addition, the number of detected lesions was considerably higher. Comparing to CT/MRI, the sensitivity of the ^68^Ga-DOTATATE PET/CT has been higher in most of the evaluated organs (30). 

Finally, in comparison with stand-alone triple-phase contrast-enhanced CT, ^68^Ga-DOTATATE PET/CT has reportedly led to the management change in 38% of the patients (34). Similar results have been documented in an evaluation of 90 patients with concordant and discordant results on triple-phase contrast-enhanced CT and ^68^Ga-DOTANOC PET/CT in which the latter has shown management alterations in overall 54.4% of the cases (35). Regarding alteration in clinical management, whole-body MRI and ^68^Ga-DOTATOC PET/CT revealed comparable results in 59% of the patients. While ^68^Ga-DOTATOC PET/CT and whole body MRI showed additional relevant findings in 31% and 14% of patients, respectively (36). In our study, comparing to CT/MRI, ^68^Ga-DOTATATE PET/CT demonstrated more involved lesions or organs in 36% of the patients, but the change of management took place in only 20% (5/25). The lower rate of management alteration in our study might be due to the utilization of both MRI and CT results rather than only one modality in most of the participants. Similar to ^99m^Tc-Octreotide SPECT/CT, the impact on clinical management was more pronounced in patients evaluated for re-staging, likely secondary to post-surgical tissue changes making the interpretation of CT/MRI more challenging. 

***Limitations***

As it is not feasible or ethical to subject all patients to multiple invasive biopsies or surgical procedures, considering the small size and location of some of the lesions, the major limitation of this study is unavailability of the histopathological tissue confirmation. The other limitation is the possibility of selection bias, caused by referral of complicated and controversial patients. Furthermore, the unavailability of Ki-67 in all patients, which is in close relation with the differentiation of tumors and SSR-imaging sensitivity, is another shortcoming of the current study. Employing different analogues of SSRs for SPECT and PET imaging (TOC vs. TATE) can also be a drawback; however, they have revealed comparable results, in previous investigations. Finally, the small number of patients is another limitation of our study.

## Conclusion

Establishment of PET-tracers targeting SSRs has evolved the functional imaging of NETs. In our study, ^68^Ga-DOTATATE PET/CT was clearly superior to ^99m^Tc-Octreotide SPECT/CT and CT/MRI showing increased sensitivity and specificity in patients with NETs. In addition, considering both ^99m^Tc-Octreotide SPECT/CT and CT/MRI results, more valuable information provided by ^68^Ga-DOTATATE PET/CT led to the management change in 24% and 20% of patients, respectively, especially in the sub-group referred for re-staging and re-evaluation for recurrence. Regarding the substantially higher cost and limitation of global availability, ^68^Ga-DOTATATE PET/CT can be particularly useful in patients demonstrating negative ^99m^Tc-Octreotide scan or CT/MRI results despite rising tumor marker levels. 
